# The Treatment of Nephritis

**Published:** 1894-08

**Authors:** J. M. Da Costa

**Affiliations:** Philadelphia


THE TREATMENT OF NEPHRITIS. (*Medical News*, April 21, 1894.) By J. M. Da Costa, M.D., LL.D., of Philadelphia.

In a typical case of recurring acute nephritis passing but five ounces of urine daily, ten drops of digitalis were administered every three hours, which, though strengthening the heart, produces no diuresis. Later, thirty grains of lactate of strontium were given four times a day, and the quantity of urine secreted on the second day thereafter was thirty-five ounces, and later, forty-eight ounces were passed in twenty-four hours, even after the digitalis had been withdrawn. The author autributes the diuretic effect produced to the use of the lactate of strontium in connection with a restricted milk diet and rest in bed.

The musical murmur which was heard was due to aortic stenosis permitting of regurgitation. The orifice was narrowed, more especially at one portion, where there was a free edge of membrane vibrating in the blood-current, or a fragment of a valve flapping to and fro.

The lactate of strontium was used in a second case of acute nephritis occurring in a cirrhotic kidney, with an increase in the quantity of urine from thirty ounces before taking the strontium to fifty-seven ounces two weeks after admission.

In third case of cirrhotic kidney the same quantity of lactate of strontium was used,—namely thirty grains daily,—and the quantity of urine, which on admission was forty ounces daily, slowly increased. The author especially advises the use of salts of strontium in *acute* renal affections.—*Ex.*

